# Pregnancy and delivery after functional hemispherectomy for Rasmussen’s encephalitis: a case report

**DOI:** 10.1186/s12883-024-03906-7

**Published:** 2024-10-23

**Authors:** Elena Jost, Waltraut M. Merz, Patrick A. Kupczyk, Laura Tascón Padrón, Eva C. Weber, Christian G. Bien, Philipp Kosian

**Affiliations:** 1 Department of Obstetrics and Prenatal Medicine, University Hospital Bonn, Venusberg-Campus 1, 53127 Bonn, Germany; 2 Department of Diagnostic and Interventional Radiology, University Hospital Bonn, Bonn, Germany; 3 Division of Prenatal Medicine, Gynecological Ultrasound and Fetal Surgery, Department of Obstetrics and Gynecology, University of Cologne, Cologne, Germany; 4 Department of Epileptology, Krankenhaus Mara, Bethel Epilepsy Center, Medical School OWL, Bielefeld University, Bielefeld, Germany

**Keywords:** Rasmussen’s encephalitis, Pre-existing condition, High-risk pregnancy, Rare disease, Case report

## Abstract

**Background:**

Rasmussen’s encephalitis (RE) is a rare neurologic disorder characterized by progressive seizures and unilateral cerebral atrophy with onset during childhood and unknown etiology. When medical therapy appears refractory, surgical disconnection of the affected hemisphere is indicated. Quality of life after functional hemispherectomy is largely good, affected females may therefore pursue pregnancy. However, data on pregnancy and delivery in RE post hemispherectomy is extremely rare.

**Case presentation:**

We present the case of a patient with left functional hemispherectomy for RE at the age of seven, who experienced two successful pregnancies. In both pregnancies, her post-surgical symptoms including right-sided spasticity, cephalgia, dizziness, and impairment of vision and speech deteriorated but improved to pre-pregnancy level after delivery. Neurologic sequelae post-hemispherectomy overlapped with clinical signs of preeclampsia and required close diagnostic surveillance during both pregnancies.

**Conclusion:**

There are no data on the interaction between RE, hemispherectomy and pregnancy, making maternal and fetal risk assessment difficult. Due to the complexity of the condition and symptoms, management of RE in pregnancy remains highly challenging and requires an interdisciplinary approach. This is the first case description of two successful pregnancies in a woman with RE and status post-hemispherectomy. Further evidence is urgently required to improve counseling and management of affected women.

## Background

Rasmussen’s encephalitis (RE) is a chronic inflammatory disorder characterized by progressive and refractory seizures, unilateral cerebral atrophy, and long-term neurologic and cognitive deficits (Orpha-ID 1929). The condition was first described by Theodor Rasmussen in 1958 [1]. In a prospective study, Bien et al. 2013 calculated an incidence of 2.4 per 10 million individuals ≤ 18 years of age [2]. The etiology is still elusive, but antigen-driven autoimmune processes mediated by cytotoxic T-cell-lymphocytes seem to be involved [3, 4].

Initial manifestations usually occur at the median age of seven years [5]. Recurrent focal seizures progress to medically refractory seizures and status epilepticus. Motor function, language (if the dominant hemisphere is affected) and cognition deteriorate [6–8].

The 2005 European consensus statement for pathogenesis, diagnosis, and treatment of RE summarized available data and established recommendations on diagnosis and management of this rare condition [7]. Diagnostic criteria encompass clinical presentation, electroencephalogram, magnetic resonance imaging (MRI), and histopathology. Treatment goals aim at minimizing seizures and cognitive-dysfunctional symptoms as well as avoiding long-term functional deterioration [9]. In addition to antiseizure medication (ASM), immunosuppressive or -modulatory treatment is available for early disease stages [10]. In case of poor seizure control and deterioration of neurologic function, surgical treatment needs to be considered. Disconnection of the affected hemisphere is performed either by anatomical or functional resection or, in more recently applied techniques, by hemispherotomy [9, 11]. Bien et al. 2009 has shown that functional hemispherectomy, which is considered a hemispherotomy, can lead to a seizure freedom rate of 70–80% [11]. Nevertheless, homonymous hemianopia and hemiplegia are inevitable sequelae of surgical treatment [9].

Studies on the long-term outcome after functional hemispherectomy reveal a good quality of life in affected individuals [12–16]. Accordingly, female patients may pursue pregnancy. The impact of pregnancy-associated alterations on patients after functional hemispherectomy are largely unknown. We report the course of two pregnancies and deliveries of a patient with RE who underwent functional hemispherectomy in early childhood. This case is reported according to CARE guidelines.

## Case presentation

### Medical and surgical history

Our patient (body weight preconception 90 kg, BMI 31,8 kg/m^2^, Northern African ethnicity) developed signs of left hemispheric RE at the age of three years. As an adopted child, detailed medical history was not available. She received various medical treatments, including sulthiame, oxcarbazepine, levetiracetam, lamotrigine, and topiramate. Additionally, she received tacrolimus during a prospective randomized trial [2]. However, progressive, drug-refractory seizures occurred; therefore, a decision for left functional hemispherectomy was taken at seven years of age. One month before surgery, presurgical monitoring revealed a slow alpha-EEG interictally with intermittent left frontal sharp-slow-waves. Two seizures were recorded within 24 h with rhythmic left frontotemporal sharp-slow-waves. MRI showed hemiatrophy on the left side including the hippocampus. A left sided Wada test demonstrated expressive and receptive verbal language capacities in the right hemisphere. A transsylvian access was chosen, the left amygdala, hippocampus and insula were resected, and the frontal, occipital and temporal lobe were dissected. Pre-surgical cognitive dysfunction, in particular motor aphasia, showed rapid improvement after surgery. Seizures subsided after surgical treatment and ASMs could be discontinued 1.5 years later. Surgical sequelae included right-sided spasticity affecting predominantly the upper limb, recurrent cephalgia, dizziness, as well as impairment of vision and speech. During puberty, recurrent depressive episodes with suicidal thoughts occurred, requiring several inpatient admissions. At the age of 17, the patient was further diagnosed with arterial hypertension due to primary hyperaldosteronism with hypokalemia, which was treated with bisoprolol (2.5 mg/d) and potassium (0.6 g/d).

### First pregnancy

The patient, then 19 years old, initially presented at our department at 23 + 1 weeks of gestation (WoG) of her first pregnancy for co-care. She did not receive preconception counselling, but transthoracic echocardiography was performed in the year preceding her pregnancy. She reported an uneventful course of pregnancy; obstetric findings including ultrasound revealed no abnormality. She was on bisoprolol (2.5 mg/d) and potassium (1.8 g/d), showing normal potassium levels under substitution.

**Fig. 1:**
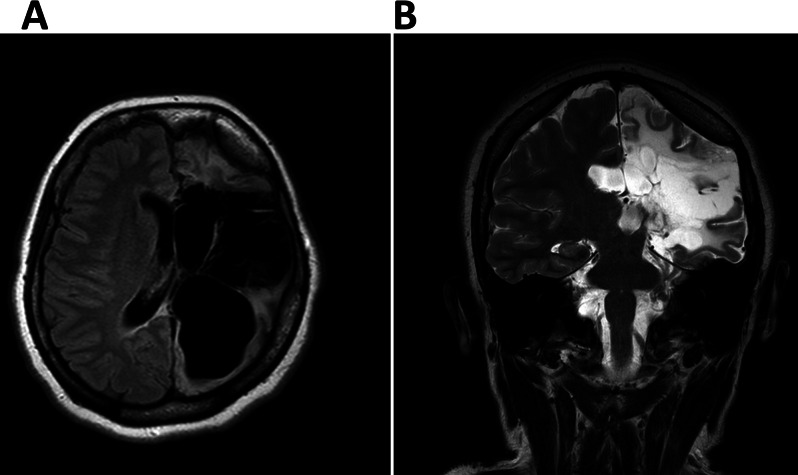
MRI showing Rasmussen’s encephalitis after left functional hemispherectomy. MRI in transverse plane (**A**, FLAIR sequence) and coronal plane (**B**, T2-TSE sequence): Extensive left hemispheric T2-hyperintense defect areas involving the cortex, ex vacuo, dilatation of the left lateral ventricle and ipsilateral midline shift reflecting unilateral cerebral atrophy

At 25 + 3 WoG, presence of vertigo for the past 4 days, nausea, vomiting, headaches, and double vision lead to hospital admission for 8 days. Laboratory results and MRI (Fig. 1) were non-contributory, excluding superimposed preeclampsia and increased intracranial pressure. Migraine was the tentative diagnosis after neurological assessment. With rising blood pressure levels, Methyldopa was added to the antihypertensive medication, and psychiatric co-care due to her known depression was initiated.

At 32 + 0 WoG our patient was readmitted for worsening vertigo and increasing blood pressure. Superimposed preeclampsia was confirmed, and close feto-maternal surveillance initiated. Within the following days, severe preeclamptic features developed, requiring delivery by cesarean section in 33 + 1 WoG. Operation and post-operative course were uncomplicated; she was discharged on postpartum day 8 in good condition. The premature neonate (female, 1750 g/25th percentile, Apgar-score 7/8/9 at 1/5/10 minutes, respectively, umbilical artery pH 7.33, base excess − 3.6 mmol/l) was transferred to the neonatal intensive care unit due to respiratory distress syndrome and prematurity. Following an uneventful course, the newborn, diagnosed with bilateral duplex kidney, could be discharged after 29 days.

### Second pregnancy

Four years after her first childbirth, then 23 years old, she presented at our department in 18 + 5 WoG of her second pregnancy due to deterioration of general condition, particularly vertigo and cephalgia.

In the inter-pregnancy interval extensive diagnostic investigations had been performed, including MRI, neurologic, ophthalmologic, and otorhinolaryngologic examinations. No information on gynecological follow-up appointments or contraception is available. Sertraline had been started for worsening depression. Again, neurologic and psychiatric co-care was initiated. Blood pressure medication was adjusted, and low-dose aspirin prophylaxis for preeclampsia was initiated. During the further course of pregnancy, multiple hospital admissions were required (five days for vaginal bleeding in 24 + 6 WoG; 18 days for deterioration of her general condition and uncertain gait in 27 + 6 WoG; and 28 days from 32 + 3 WoG onwards). Elective repeat cesarean section with bilateral tubal ligation upon patient’s request due to deterioration of general condition was performed in 36 + 2 WoG, with delivery of a healthy, premature neonate (female, 3210 g/83rd percentile, Apgar-score 7/9/10 at 1/5/10 min, respectively, umbilical artery pH 7.20, base excess − 5.30 mmol/l). After uneventful intra- and postoperative course, the patient and her newborn could be discharged on day 3 postpartum.

## Discussion

We report on pregnancy and delivery in a patient 12 and 16 years after left functional hemispherectomy for RE. Our case illustrates typical challenges that emerge in the care of women with rare diseases during pregnancy and delivery. Most importantly, interactions between pregnancy and the pre-existing condition are unknown, rendering predictions on the course of pregnancy, risk of complications, and potential impacts on the pre-existing condition impossible.

Our patient had several risk factors for developing preeclampsia, namely pre-existing hypertension, ethnicity, parity, age and weight [17]. Her post-hemispherectomy complaints consisted, among others, of headaches and visual disturbance. These symptoms are characteristic features of preeclampsia. Since her post-hemispherectomy symptoms deteriorated during course of pregnancy, distinction to superimposed preeclampsia became a diagnostic challenge. Close surveillance with serial feto-maternal controls and interdisciplinary cooperation was therefore necessary.

Fortunately, serious maternal morbidity [18] did not occur, and she recovered fully after delivery in both pregnancies. With respect to the perinatal outcome, even though both children were born preterm, they did not suffer serious neonatal complications and are healthy. For other autoimmune-related diseases such as multiple sclerosis, an elevated recurrence risk in offspring is described [19]. Since the etiology of RE is still unknown, their risk to develop RE cannot be quantified. Familial cases have not been noted so far.

Current research on the pathophysiology of RE suggest autoimmune mechanisms [3, 7]. Pregnancy is known for its influence on the immune system due to hormonal changes and the need for immunologic tolerance of fetal antigens [20]. Interactions between autoimmune diseases and pregnancy however are diverse and include improvement as well as deterioration [21, 22]. Levin et al. 2020 reported increased rates of miscarriage but normal neonatal outcome in case of livebirths in women suffering from autoimmune encephalitis. However, RE is not specified and transfer of evidence from other conditions may be erroneous [23].

We could not identify any report on pregnancy and delivery in women after hemispherectomy for RE. Larner et al. 1995 described a case of first occurrence of seizures in a 22-year-old patient during early pregnancy and postpartum, followed by establishment of RE diagnosis. Obstetric and neonatal outcome was not documented [24]. Maralit et al. 2017 portrayed the case of a 34-year-old woman with establishment of RE diagnosis during pregnancy. She presented at 29 WoG in her fifth pregnancy with seizures, hemiparesis, and cognitive deficits. MRI demonstrated unilateral cerebral hemiatrophy. Treatment was initiated during pregnancy and she delivered a preterm healthy neonate [25]. Both cases illustrate late-adult-onset RE, which has been described to occur in up to 10% of RE [5, 6, 26, 27].

We identified one case report describing two successful pregnancies in a 31-year-old woman after hemispherectomy during childhood for Sturge-Weber syndrome. She gave birth vaginally to two healthy newborns [28].

To the best of our knowledge, this is the first case to describe two successful pregnancies in a woman after functional hemispherectomy for RE. With this work, we aim at contributing to the sparse evidence on the course of this condition during pregnancy, childbirth and postpartum to improve preconception counseling and maternity care for affected women.

## Conclusion

Pregnancies in women with rare conditions are often fraught by a lack of knowledge about possible interactions, maternal, fetal, and perinatal consequences, and complications, as well as long-term sequelae for both, mother and child. An interdisciplinary, individualized management approach is essential. Further reports on pregnancies in women with rare diseases are urgently required to increase evidence and improve care of affected women.

## Data Availability

No datasets were generated or analysed during the current study.

## Abbreviations

ASM: Antiseizure medication
MRI: Magnetic resonance imaging
RE: Rasmussen’s encephalitis
WoG: Weeks of gestation
